# Determinants of modern contraceptive utilization among married women in sub-Saharan Africa: multilevel analysis using recent demographic and health survey

**DOI:** 10.1186/s12905-022-01769-z

**Published:** 2022-05-18

**Authors:** Zemenu Tadesse Tesema, Getayeneh Antehunegn Tesema, Moges Muluneh Boke, Temesgen Yihunie Akalu

**Affiliations:** 1 Department of Epidemiology and Biostatistics, Institute of Public Health, College of Medicine and Health Sciences, University of Gondar, Gondar, Ethiopia; 2 Department of Reproductive Health, Institute of Public Health, College of Medicine and Health Sciences, University of Gondar, Gondar, Ethiopia

**Keywords:** Modern contraception, Family planning, Sub-Saharan, Multilevel analysis

## Abstract

**Background:**

Family planning is a low-cost, high-impact public health and development strategy to improve child and maternal health. However, there is a lack of evidence on modern contraceptive use and determinants in sub-Saharan Africa. Hence, this study aimed at determining the pooled prevalence and determinants of modern contraceptive utilization among married women of sub-Saharan Africa.

**Methods:**

Thirty-six sub-Saharan African countries' demographic and health survey (DHS) data were used for pooled analysis. A total weighted sample of 322,525 married women was included. Cross tabulations and summary statistics were done using STATA version 14 software. The pooled prevalence of modern contraceptive utilization with a 95% Confidence Interval (CI) was reported. Multilevel regression analysis was used to identify the determinants of modern contraceptive use among married women. Four models were fitted to select the best-fitted model using the Likelihood Ratio (LLR) and Deviance test. Finally, the model with the highest LLR and the smallest deviance was selected as the best-fitted model.

**Results:**

The pooled estimate of modern contraception use in sub–Saharan African countries was 18.36% [95% CI: 18.24, 18.48], with highest in Lesotho (59.79%) and the lowest in Chad (5.04%). The odds of modern contraception utilization were high among women living in East Africa [AOR = 1.47 (1.40, 1.54)], urban areas [AOR = 1.18 (1.14, 1.24)], and women with primary [AOR = 1.49 (1.44, 1.55)] and secondary and above educational level [AOR = 1.66 (1.58, 1.74)]. Moreover, husbands with primary educational level [AOR = 1.38 (1.33, 1.42)], middle [AOR = 1.17, (1.14, 1.21)], rich wealth status [AOR = 1.29 (1.25, 1.34)], media exposure [AOR = 1.25 (1.22, 1.29)], and postnatal care (PNC) utilization [AOR = 1.25 (1.22, 1.29)] had higher odds of modern contraceptive utilization compared with their counter parts. Furthermore, deliver at health facility [AOR = 1.74 (1.69, 1.79)] and birth order 2–4 [AOR = 1.36 (1.31, 1.41)] had higher odds of modern contraceptive utilization. On the other hand, women living in Central [AOR = 0.23 (0.22, 0.24)], Western regions [AOR = 0.46 (0.40, 0.54)], women who decided with husband [AOR = 0.90 (0.87, 0.93)], and decisions by husband alone [AOR = 0.73 (0.71, 0.75)] decreased the odds of modern contraceptive utilization.

**Conclusion:**

The uptake of modern contraception in sub-Saharan Africa is low. Modern contraceptive utilization is affected by different factors. More attention needs to be given to rural residents, illiterate women, and communities with low wealth status.

## Background

Contraception is the way to prevent pregnancy and assists couples and individuals to achieve the reproductive goals that enable them to exercise their reproductive rights, spacing of birth interval, limit the number of children, and reduces maternal and child morbidity and mortality related to complications of unwanted pregnancy [1]. In low and middle-income countries, the burden of maternal mortality ratio (MMR) is projected up to 415 maternal deaths per 100,000 live births, which is sixty times higher than the high-income countries. Sub-Saharan Africa is the leading region in MMR. One woman will die in every 37 women in Sub-Saharan Africa compared to developed countries’ 1 in 7800 [2].

In most sub-Saharan countries, the total fertility rate (TFR) is declined. However, in some countries, TFR remains stagnant. Countries like Zimbabwe, Namibia, Liberia, Kenya, Togo, Senegal, Madagascar, and Ghana showed the steepest drop in total fertility. Niger, Nigeria, Mozambique, and other countries remain the same or increased [3]. Improving contraception utilization is essential to accelerate the fertility decrement rate in sub-Saharan countries [4].

Currently globally, 1.1 billion women need family planning, 851 million women use a modern method of contraception, 85 million use a traditional method, and 172 million women have an unmet need for contraception [5]. World reproductive age women (15–49) using contraception increased in the last decades, but the progress was uneven between regions. Contraceptive use in Latin and North America, Asia, and Australia range from 60 to 50%, in contrast, Northern Africa and Western Asia (35.1%), sub-Saharan Africa (34.1%), and Oceania (30.7%) had few numbers of women use contraception [6].

Evidence suggests that modern contraceptive usage among married women in sub-Saharan African countries ranges from 4 to 52% [7–9]. Socio-demographic characteristics like age, residence, education level, religion, level of income, marital status, employment, obstetric history-related variables such as parity, number of living children, number of antenatal visits, prior HIV testing, knowledge of methods, and husband involvement has been reported as determinants of contraceptive utilization[10–14].

Expanding contraception access and ensuring family planning met through using effective contraceptive methods are critical steps toward attaining many of 17 Sustainable Development Goals (SDG) and 169 targets of the 2030 agenda related to universal access to sexual and reproductive healthcare services, gender equality, maternal and child health, nutrition, and women’s and girl’s empowerment [15].

To improve the coverage of contraception in Sub-Saharan Africa, strengthening healthcare systems with commodity supply, integrating family planning service with other health care services, strengthening commercial outlet, increasing the knowledge of clients, encouraging male involvement, free accessing or reducing the price of contraception methods, and training health care providers on counseling and the technical procedure has been done for last two decades [16, 17]. Despite the effort to enhance the coverage of modern contraception, there is a limitation of representative primary data source that provides prevalence and determinants to contraception use in sub-Saharan Africa. According to a recent report, the data gap is the main challenge to tracking the SDG index in Africa [18]. Therefore, this study seeks to determine the pooled prevalence and determinants of modern contraceptive utilization across sub-Saharan African countries.

## Method

### Data source

The most recent (from 2006 to 2018) Demographic and Health Surveys (DHS) data were used in the following 36 sub-Saharan African countries (Table 1). These datasets were used to determine the pooled prevalence and determinants of modern contraception use across countries in sub-Saharan Africa. The DHS is a national survey that collects information on basic demographic and health indicators such as family planning service use, mortality, morbidity, and mother and child health and fertility. The information came from the DHS measure program. Men, women, children, birth, and household datasets are included in each country's survey; the IR file was employed for this study. Women's data set was used to extract both dependent and independent variables. A two-stage stratified selection procedure was done to select study participants. The study contained a total weighted sample of 322,525 married women from 36 DHS surveys conducted in sub-Saharan African nations in the five years before the survey.

**Table 1:** Pooled Demographic and Health Surveys (DHS) data from 36 sub-Saharan countries, 2006–2018

Country	DHS year	Sample size (322,525)
**Southern Region of Africa**		**16,071**
Lesotho	2014	3612
Namibia	2013	3120
Swaziland	2006/07	6289
South Africa	2016	3049
**Central Region of Africa**		**55,588**
Angola	2015/16	7956
DR Congo	2013/14	12,095
Congo	2011/12	6289
Cameroon	2011	9791
Gabon	2012	4474
Sao Tome & Principe	2008/09	1718
Chad	2014/15	13,262
**Eastern region of Africa**		**119,530**
Burundi	2010	9781
Ethiopia	2016	10,223
Kenya	2014	18,549
Comoros	2012	3261
Madagascar	2008/09	12,038
Malawi	2015/16	16,130
Mozambique	2011	9331
Rwanda	2014/15	6981
Tanzania	2015/16	8210
Uganda	2011	11,223
Zambia	2018	7648
Zimbabwe	2013/14	6151
**Western Region of Africa**		**131,334**
Burkina-Faso	2010	13,563
Benin	2017	11,168
Cote d’Ivoire	2011	6308
Ghana	2014	5321
Gambia	2013	6791
Guinea	2018	7727
Liberia	2013	5385
Mali	2018	8567
Nigeria	2018	29,089
Niger	2012	9880
Sierra Leone	2010/11	10,902
Senegal	2010/11	10,346
Togo	2013/14	6281

### Measurements of variables

#### Outcome variable

The outcome variable for this study was whether a mother used modern contraceptives or not. The outcome of this study was binary (modern contraceptive use versus non-modern contraceptive). We coded “1″ if women utilized modern contraceptives and”0″ otherwise.

#### Explanatory variables

Based on known facts and literatures the explanatory variables included in this study were Region, residence, age group, literacy level, maternal education, husband education, maternal occupational status, women's autonomy on health care, wealth index, media exposure, accessing health care, PNC utilization, place of delivery and birth order[19–22].

#### Data management and analysis

We integrated data from 36 nations in Sub-Saharan Africa after extracting variables based on literature. Before conducting the statistical analysis, the data was weighted to keep the representativeness of the survey and to get more reliable estimates. STATA version 14 was used to perform cross-tabulations and summary statistics. Moreover, the pooled prevalence of modern contraceptive utilization with a corresponding 95% confidence interval (CI) was reported.

#### Statistical modeling

The demographic and health survey data has a hierarchical structure for the factors, which violates the usual logistic regression model's assumption; independence of observations and equal variance. Besides, women within a single cluster are expected to be more similar than other clusters in the country. Hence, using multilevel analysis is recommended to take into account the between cluster variability. To address this objective four separate models were fitted. These include the null model, model I (community-level variables), model II (individual-level variable), and Model III (models that include both individual and community level variables) were fitted. The most parsimonious model was selected using LLR and Deviance test. The highest LLR and the lowest deviance was the best-fitted model. Finally, model III, which included both individual and community level variables, were selected (Table XXX3).

## Results

A total of 322,525 married women five years preceding the survey in 36 sub-Saharan African countries were included in this study. Of these, the largest study participants 131,334 (40.72%) were from Western Africa Region and the smallest study participants 16,071 (4.98%) were from the Southern Regions of Africa. The majority of study participants 212,360 (65.84%) were rural residents. The median age of women included in his study was 28.8 (IQR = 7.2) years of which 129,302 (40.09%) of them were aged 25–34. Thirty-seven percent of women and thirty-nine percent of men had no formal education. More than one-third of women 127,562 (39.55%) were within the poor wealth status (Table 2).

**Table 2:** Distribution of modern contraceptive utilization in sub-Saharan Africa region

Variable	Modern contraceptive utilization		Total (%)	X-square value	*p*-value
	Yes	No			
**Africa region**					
Southern	6789	9282	16,071 (4.98)	163.79	< 0.001
Central	6728	48,860	55,588 (17.24)		
Eastern	47,646	71,883	119,530 (37.66)		
Western	17,936	11,3397	131,334 (40.72)		
**Residence**					
Rural	47,359	16,5001	212,360 (65.84)	46.19	< 0.001
Urban	31,742	78,422	110,165 (34.16)		
**Age group**					
15–24	16,472	62,396	78,868 (24.45)	20.08	< 0.001
25–34	35,393	93,908	129,302 (40.09)		
35–46	27,235	87,119	114,355 (35.46)		
**Literacy**					
Cannot read and write	23,,822	140,763	164,585 (51.03)	103.73	< 0.001
Can read and write	55,279	102,660	157,940 (48.97)		
**Maternal education**					
No education	15,384	111,585	126,969 (39.37)	103.73	< 0.001
Primary education	33,176	72,974	106,151 (32.91)		
Secondary and above	30,541	58,861	89,405 (27.72)		
**Husband education**					
No education	13,905	98,391	112,296 (35.95)	70.97	< 0.001
Primary education	26,193	60,678	86,871 (27.81)		
Secondary and above	33,694	79,478	113,173 (36.24)		
**Maternal occupation**					
Had occupation	60,594	182,407	243,001 (75.34)	152.34	< 0.001
Had no occupation	18,507	61,017	79,524 (24.66)		
**Women’s health care decision making autonomy**					
Women alone	17,865	38,350	56,216 (17.43)	118.50	
Women and her husband	33,837	84,186	118,024 (36.59)		< 0.001
Husbands alone	27,398	120,886	148,284 (45.98)		
**Wealth Index**					
Poor	23,500	104,061	127,562 (39.55)	124.50	< 0.001
Middle	15,318	48,697	64,015 (19.85)		
Rich	40,282	90,665	130,947 (40.60)		
**Media exposed**					
Yes	61,967	158,840	220,807 (68.46)	112.47	< 0.001
No	17,134	84,583	101,718 (31.54)		
**Accessing health care**					
Big problem	40,513	143,803	184,316 (57.15)	38.16	< 0.001
Not big problem	38,588	99,620	138,209 (42.85)		
**PNC utilization**					
Yes	26,673	57,748	113,799 (57.41)	62.26	< 0.001
No	23,777	90,021	8441 (42.59)		
**Place of delivery**					
Home	11,266	69,960	75,227 (31.26)	136.58	< 0.001
Institution	49,199	116,214	165,413 (68.74)		
**Birth order**					
1	11,661	35,991	47,652 (14.81)	68.29	< 0.001
2–4	41,817	101,145	142,963 (44.43)		
5+	25,440	105,698	131,138 (40.76)		
**Children ever born**					
0	1,544	21,044	22,588 (7.00)	92.39	< 0.001
1–3	41,890	107,871	149,761 (46.43)		
4+	35,667	114,508	150,175 (46.56)		

### The pooled prevalence of modern contraception utilization

**Fig. 1:**
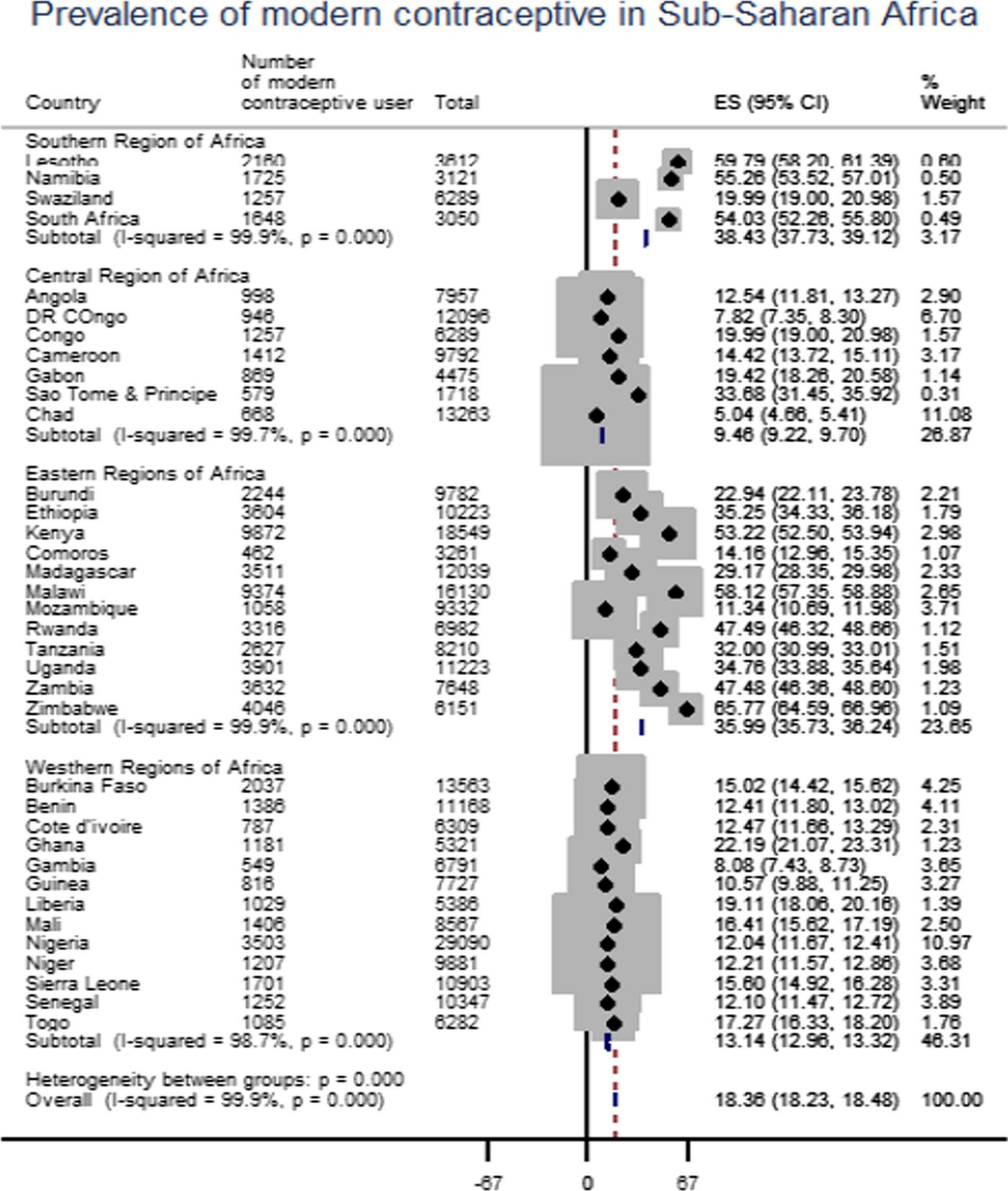
Forest plot of modern contraceptive utilization in Sub-Sahara Africa from 2006 to 2018

The pooled prevalence of modern contraceptive utilization in sub-Saharan Africa countries was 18.36% [95% CI: 18.24, 18.48], with the highest modern contraceptive utilization in the Southern Region of Africa (38.43%) and the low modern contraceptive utilization in Central Regions of Africa (9.46%). The sub-group analysis result evidenced that in the Southern regions of Africa highest modern contraceptive utilization (59.79%) was recorded in Lesotho and the low modern contraceptive utilization (19.99%) was recorded in Swaziland. In the Central Regions of Africa highest modern contraceptive utilization (33.68%) was recorded in Sao Tome and Principe and the low modern contraceptive utilization (5.04%) was recorded in Chad. In Eastern regions of Africa highest modern contraceptive utilization (65.77%) was recorded in Zimbabwe and the low modern contraceptive utilization (11.34%) was from Mozambique. In the Western Regions of Africa, the highest modern contraceptive utilization (22.19%) was from Ghana and the low contraceptive utilization (8%) was from Gambia (Fig. 1).

### Determinants of modern contraceptive utilization

#### Random effect analysis

The results of the random-effects model indicated that there was significant clustering of modern contraceptive utilization across the communities (OR of community-level variance = 0.03, 95% CI = 0.024–0.038). The intra-class correlation (ICC) in the null model indicated that 5.13% of the overall variability of modern contraceptive utilization was attributed to cluster variability.

The median odds ratio (MOR) for modern contraceptive utilization was 1.49 in the null model, which indicates that there was a variation in modern contraceptive utilization between clusters. This means if we randomly select women from different clusters, women at the cluster with higher modern contraceptive utilization had 1.49 times higher odds of modern contraceptive utilization as compared with those women at clusters with lower modern contraceptive use.

The Proportional Change in Variance (PCV) also increases from 80% from model II to 83% in model III (a model with individual and community level variables), which indicates the last model (model III) best explains the variability of modern contraceptive utilization (Table 3).

**Table 3:** Determinants of modern contraceptive uptake among married women in sub-Saharan Africa

Variable	Null model AOR (95% CI)	Model I AOR (95% CI)	Model II AOR (95% CI)	Model III AOR (95% CI)
**Africa Region**				
Southern		1		1
Central		0.17 (0.16, 0.17)		0.23 (0.22, 0.24)*
Eastern		1.00 (0.96, 1.03)		1.47 (1.40, 1.54)*
Western		0.23 (0.22, 0.24)		0.46 (0.43, 0.48)*
**Residence**				
Rural		1		1
Urban		1.77 (1.73, 1.83)		1.18 (1.14, 1.24)*
**Age group**				
15–24			1	1
25–34			1.06 (1.02, 1.08)	0.99 (0.96, 1.02)
35–46			0.99 (0.95, 1.04)	0.92 (0.88, 1.01)
**Maternal education**				
No education			1	1
Primary education			1.62 (1.57, 1.68)	1.49 (1.44, 1.55)*
Secondary and above			1.43 (1.36, 1.49)	1.66 (1.58, 1.74)*
**Husband education**				
No education			1	1
Primary education			1.84 (1.78, 1.90)	1.38 (1.33, 1.42)*
Secondary and above			1.36 (1.31, 1.41)	1.38 (1.33, 1.43)*
**Maternal occupation**				
Had no occupation			1	1
Had occupation			0.86 (0.84, 0.88)	0.94 (0.91, 1.01)
**Women’s health care decision making autonomy**				
Women alone			1	1
Women and her husband			0.81 (0.78, 0.83)	0.90 (0.87, 0.93)*
Husbands alone			0.50 (0.48, 0.51)	0.73 (0.71, 0.75)*
**Wealth Index**				
Poor			1	
Middle			1.14 (1.10, 1.17)	1.17 (1.14, 1.21)*
Rich			1.35 (1.31, 1.38)	1.29 (1.25, 1.34)*
**Media exposed**				
No			1	1
Yes			1.21 (1.18, 1.24)	1.25 (1.22, 1.29)*
**Accessing health care**				
Big problem			1	1
Not big problem			1.07 (1.04, 1.09)	1.03 (0.98, 1.05)
**PNC utilization**				
No			1	1
Yes			1.59 (1.56, 1.63)	1.62 (1.58, 1.66)*
**Place of delivery**				
Home			1	1
Institution			1.90 (1.83, 1.95)	1.74 (1.69, 1.79)*
**Birth order**				
1			1	1
2–4			1.26 (1.22, 1.31)	1.36 (1.31, 1.41)*
5+			1.20 (1.14, 1.27)	1.35 (1.28, 1.43)*
Community Variance (SE)	0.17 (0.15, 0.20)	0.068 (0.05, 0.08)	0.035 (0.028, 0.044)	0.030 (0.024, 0.038)
ICC%	5.13	2	1.5	1.4
PCV%	1	88.23	80.22	83.05
MOR	1.49 (1.42, 1.53)	1.28 (1.23, 1.30)	1.19 (1.17, 1.22)	1.17 (1.15, 1.19)
LL	−175,175	−158,668	−97,036	−91,641
Deviance	350,350	317,336	194,072	183,282
AIC	350,354	317,349	194,114	183,332
BIC	350,376	317,413	194,329	183,587

1 = reference, * = *p* < 0.05

In the multilevel multivariable logistic regression model; Sub-Sahara Africa region, residence, maternal education, husband education, women health care decision autonomy, wealth index, media exposure, PNC utilization, place of delivery, and birth order were identified as determinants to modern contraceptive utilization.

Women living in Central, and Western Regions of Africa decreased the odds of modern contraceptive utilization by 77%, and 54% as compared to women living in South Regions of Africa (AOR = 0.23, 95% CI: 0.22, 0.24), and (AOR = 0.46, 95% CI: 0.40, 0.54), respectively. The odds of modern contraceptives were increased by 47% among women living in East Africa as compared to women living in South Africa (AOR = 1.47, 95% CI: 1.40, 1.54). The odds of modern contraceptive utilization among urban women were increased by 18% as compared to rural women (AOR = 1.18, 95% CI: 1.14, 1.24).

The odds of modern contraceptive utilization among women who had primary and secondary and above educational level were 1.49 (AOR = 1.49, 95% CI: 1.44, 1.55) and 1.66 (AOR = 1.66, 95% CI: 1.58, 1.74) times higher as compared to women who had no formal education. The odds of modern contraceptive utilization among women whose husbands had primary and secondary and above educational level were 1.38 (AOR = 1.38, 95% CI: 1.33, 1.42) and 1.38 (AOR = 1.38, 95% CI: 1.36, 1.47) times higher as compared to women whose husband had no formal education. The odds of modern contraceptive utilization among women who can decide health care service with their husband and husband alone were decreased 10% (AOR = 0.90, 95% CI: 0.87, 0.93) and 27% (AOR = 0.73, 95% CI: 0.71, 0.75) as compared to women whose health care utilization decided by herself alone.

Women with middle and rich wealth status were 1.17 (AOR = 1.17, 95% CI: 1.14, 1.21) and 1.29 (AOR = 1.29, 95% CI: 1.25, 1.34) times more likely to utilize contraceptives than poor women. The odds of contraceptive utilization among media-exposed women were 1.25 times higher than women who were not exposed to media (AOR = 1.25, 95% CI: 1.22, 1.29). The odds of modern contraceptive utilization among women who had PNC utilization were 1.62 times more likely than women who had no PNC utilization (AOR = 1.25, 95% CI: 1.22, 1.29). The odds of modern contraceptive utilization among women who deliver at the health facility were 1.62 times more likely than women who deliver at home (AOR = 1.74, 95% CI: 1.69, 1.79). The odds of contraceptive utilization among women whose birth order 2–4 and 5 + were increased by 36% (AOR = 1.36, 95% CI: 1.31, 1.41) and 35% (AOR = 1.35, 95% CI: 1.28, 1.43) as compared to women who had first birth order (Table 3).

## Discussion

In this study, the pooled prevalence of modern contraceptives was 18.36% (18.23–18.48%). Socio-demographic factors (residency, maternal education, husband education), the person who provides health care decision making, wealth index, media exposure, obstetric history-related factors such as PNC utilization, place of delivery, and birth order were significantly associated with modern contraceptive utilization in Sub-Saharan Africa.

The highest modern contraceptive utilization was found in the southern Africa region (38.43%) and the lowest in the central Africa region (9.46%). This study showed that in the southern Africa region the highest prevalence was noticed in Lesotho (59.79%) and the lowest in Swaziland (19.99%). In the Eastern Africa region, the overall modern contraceptive prevalence was 35.99% with the highest and lowest prevalence was found in Zimbabwe (65.77%) and Mozambique (11.34%), respectively. The overall modern contraceptive in the western Africa region was 13.14%. Of these, the highest prevalence was found in Ghana (22.19%) and the lowest prevalence of modern contraceptives was found in Gambia (8.08%). The highest prevalence in central Africa (19.99% in Congo) was lower than the lowest prevalence in many other countries.

This finding is in line with a recent review performed in the SSA [23]. Besides, the current study is in line with a study from the 2018 World Health Organization (WHO) report that showed that 5.4 children were born from a mother [24]. This indicates the uptake of modern contraceptives in the SSA region is still low. This could be because of repeated conflicts and security issues, the need for a large family, fear of side effects like infertility, religious and cultural restrictions [21], sex preference, spouse consent and support, high level of illiteracy, poverty, and health system barriers in the sub-Saharan Africa region [23]. Even though the possible barriers are perfectly preventable, measures applied to ensure adequate uptake of modern contraceptive in the SSA was very limited [25]. Thus, it is of the essence to all SSA countries to ensure the implementation of adequate sustainable measures to increase the uptake of modern contraception. As a result, they will guarantee sustainable global development, poverty alleviation, increased life expectancy, empowering of women, promoting of health through the reduction of maternal mortality, morbidity, unsafe abortion, and improve child survival through birth spacing [26, 27].

The finding of the current study result is low than the SDG target (75%) [28]. A rise in the proportion of women of reproductive age who use modern contraception to meet their family planning needs (SDG indicator 3.7.1) will help in achieving other 2030 Agenda goals and targets, such as lowering maternal mortality (SDG 3.1.1) and under-5 child mortality (SDG 3.1.2), increase educational attainment (SDG 4.3.1), and to reduce the number of women and children living in poverty (SDG 1.2.1) [29].

The African region, residency, maternal education, husband education, the person who provides health care decision making, wealth index, media exposure, PNC utilization, place of delivery, and birth order were significantly associated with modern contraceptive utilization in Sub-Saharan Africa.

The odds of having modern contraceptive were low in the central, eastern, and western African region compared with the southern African region. This study is in agreement with a trend analysis carried out in SSA countries between 1990 and 2014 [30]. According to the findings of the former study Southern African region had a faster increase in contraceptive prevalence rate with some countries achieving almost 60% and trends in completed family size is the lowest compared with other African regions. In contrast, the Central Africa region has a steady contraceptive rate and the lowest across the period and had the highest total fertility rate, completed family size, and family size preference. As a result, the progression of modern contraceptive utilization in the central Africa region had a very slow progression increment with many below 20% as of 2014 [31]. The possible reason could be a poor health care system in the central Africa region. For instance, a study from Nigeria, Ghana, and Kenya showed that health care indicators including user-fees, type of health facility, visit by a health care worker, adolescent reproductive health, regular availability of health care workers, and the number of professionals working on maternal health would highly valuable in the utilization of modern contraceptive use [32].

Living in an urban area was associated with a better modern contraceptive utilization compared with the rural counterparts. The possible reason could be women in rural areas had poor service availability and accessibility and they are far from the health facility [22]. Besides, women in the rural area were more likely uneducated and they are unable to get the method of their choice.

In this study, maternal and husband education increases the uptake of modern contraceptives. This finding is in line with a systematic review and meta-analysis carried out in SSA countries between 2005 and 2015 [19]. Similarly, trend analysis in contraceptive prevalence in SSA showed that women's education was strongly correlated with the high level of contraceptive uptake [30]. This could be due to the fact that education is the power and a precursor to developing women's empowerment through improving their knowledge and attitude [33]. Also, education creates a good job opportunity or employment that could cause child spacing [34] which further improves child survival [35]. However, the former study revealed that the mere presence of female education was not adequate and it is highly recommended emphasizing the presence of voluntarily family planning services was strongly associated with an increase in the prevalence of contraceptive use.

Women’s health care decision-making autonomy was significantly affecting the odds of modern contraceptive uptake. Women who decide with their husbands had higher odds of modern contraceptive uptake than women who decide alone. This could be when women are unable to decide by themselves their rights and the ability to choose and use the method was worse. Empowering women would enhance women’s decision-making ability and increasing their knowledge level that can further improve modern contraceptive uptake [36]. As a result, the maternal health outcome and child health would be improved in Sub-Saharan Africa.

The odds of having modern contraceptive uptake were higher among women with a higher wealth index. This finding was supported by a cross-sectional study conducted in sub-Saharan African countries using Kenya and Zimbabwe demographic and health survey data [37]. The possible explanation could be women from middle and high wealth index have better financial resources which are very helpful to get better access to reproductive health services including modern contraceptive uptake [38].

Mass media exposure was associated with higher odds of modern contraceptive uptake. This finding was supported by a systematic review and meta-analysis conducted among 31 sub-Saharan African countries using 47 demographic and health surveys conducted between 2005 and 2015 [39]. The possible reason could be mass media exposure can expose people to information [40, 41] and overcome barriers of illiteracy and improve the knowledge and attitude of women [20].

The current study showed that mothers who had PNS services had higher odds of utilizing modern contraceptives. The possible reason could be during PNC follow-up mothers’ have the opportunity to communicate with providers and to receive counseling regarding on initiation of postpartum contraceptive services [42, 43]. Similarly, mothers’ who delivered at health institution has higher odds of uptake of modern contraceptive. This is because at the health institution mothers’ have the opportunity to get information on when and why they initiate postpartum contraception [44]. Birth orders of two or more had higher odds of modern contraceptive intake than birth order one. This because women’s having more children had the intention to have birth spacing than women’s having a single child or fewer children.

The strength of the current study was incorporating 36 sub-Saharan Africa countries and the findings can be easily generalized to the SSA at large. However, it is difficult to establish a temporal relationship because of the cross-sectional nature of the study. Moreover, data related to the availability and accessibility of the service was not collected. Lastly, the study will be prone to recall and social desirability bias as most of the health measures in DHS are based on self-report.

The uptake of modern contraceptive utilization has a valuable contribution to the general public and the country at large. As a result, the public will be benefit by maintaining health promotion including reduction of poor maternal outcomes (maternal morbidity, mortality, and abortion) and in poverty mitigation, women empowerment, increased life expectancy, keeping gender equality, and realizing sustainable global development goals. Besides, child survival will be improved through birth spacing and has a great contribution in creating a better future for the coming generations.

## Conclusion

This study indicated low modern contraception utilization in sub-Saharan Africa. Women living in the east, central, western region, urban, educational level, wealth status, media exposure, having PNC utilization, delivering at the health facility, and having more birth order, women's decision-making ability were significantly associated with modern contraception utilization. More attention needs to be given to rural residents, illiterate women, and communities with low wealth status. Moreover, postnatal care and health facility delivery activities need to be strengthened.

## Data Availability

Data is available online on www.measuredhs.com**.**

## Abbreviations

AIDS: Acquired Immune Deficiency Syndrome
AOR: Adjusted Odd Ratio
CBD: Community-Based Distribution
CI: Confidence Interval
COR: Crude Odd Ratio
DHS: Demographic and Health Survey
ICC: Intra-Class Correlation
HIV: Human Immune Virus
IQR: Inter Quartile Range
LLR: Likelihood Ratio
MMR: Maternal Mortality Ratio
MOR: Median Odds Ratio
PCV: Proportional Change in Variance
PNC: Postnatal care
SSA: Sub-Saharan Africa
TFR: Total Fertility Rate
